# ASD^2^-TL∗ GTO: Autism spectrum disorders detection via transfer learning with gorilla troops optimizer framework

**DOI:** 10.1016/j.heliyon.2023.e21530

**Published:** 2023-11-02

**Authors:** Abdulqader M. Almars, Mahmoud Badawy, Mostafa A. Elhosseini

**Affiliations:** a Taibah University, College of Computer Science and Engineering, Yanbu, 46421, Saudi Arabia; b Taibah University, Applied College, Computer Science, and Information Department, Medina, 41461, Saudi Arabia; c Mansoura University, Faculty of Engineering, Computers and Control Systems Engineering Department, Mansoura, 35516, Egypt

**Keywords:** Autism, Autistic children dataset, Deep learning (DL), Gorilla troops optimizer, Machine learning (ML), Pretrained model

## Abstract

Autism Spectrum Disorder (ASD) treatment requires accurate diagnosis and effective rehabilitation. Artificial intelligence (AI) techniques in medical diagnosis and rehabilitation can aid doctors in detecting a wide range of diseases more effectively. Nevertheless, due to its highly heterogeneous symptoms and complicated nature, ASD diagnostics continues to be a challenge for researchers. This study introduces an intelligent system based on the Artificial Gorilla Troops Optimizer (GTO) metaheuristic optimizer to detect ASD using Deep Learning and Machine Learning. Kaggle and UCI ML Repository are the data sources used in this study. The first dataset is the Autistic Children Data Set, which contains 3,374 facial images of children divided into Autistic and Non-Autistic categories. The second dataset is a compilation of data from three numerical repositories: (1) Autism Screening Adults, (2) Autistic Spectrum Disorder Screening Data for Adolescents, and (3) Autistic Spectrum Disorder Screening Data for Children. When it comes to image dataset experiments, the most notable results are (1) a TF learning ratio greater than or equal to 50 is recommended, (2) all models recommend data augmentation, and (3) the DenseNet169 model reports the lowest loss value of 0.512. Concerning the numeric dataset, five experiments recommend standardization and the final five attributes are optional in the classification process. The performance metrics demonstrate the worthiness of the proposed feature selection technique using GTO more than counterparts in the literature review.

## 1 Introduction

Behavioral, social, and communication impairments are the hallmarks of autism spectrum disorder (ASD) [1]. Repeated behaviors and delays in motor skill development are also symptoms of ASD [2]. These diseases can generally be distinguished from three years of age using available diagnostic protocols. ASD usually manifests in the first two years; the symptoms last a lifetime. Autism affects many parts of the brain. The gene interactions or polymorphisms contributing to this disorder also affect it genetically. ASD affects approximately one child out of 70 worldwide. The US CDC [3] stated that 168 out of 10,000 children were diagnosed with ASD in 2018, the highest rate on record. Boys have a higher prevalence rate of ASD than girls. The United States has an estimated 3.63 % of boys with ASD aged 3–17, while girls have an estimated 1.5 %.

There is no specific treatment for ASD, but various treatment approaches have been devised to alleviate symptoms and improve cognitive abilities, daily life skills, and functionality in individuals with ASD [2]. Early treatment, however, can significantly improve the symptoms and functional abilities. The most common intervention methods for patients who have ASD are behavioral and cognitive, with some relying on evolutionary approaches. Early intervention can improve social skills, interaction, and neurodevelopment for individuals with autism spectrum disorder (ASD). To achieve this, it is necessary to develop an effective and precise method of diagnosing ASD. However, ASD diagnosis can be challenging due to its complex and heterogeneous symptoms. Therefore, manual screenings are tedious, time-consuming, and susceptible to human error.

As a branch of artificial intelligence, machine learning (ML) is a technique that enables computers to automatically analyze large datasets and find patterns to make decisions about them. Supervised ML can predict a given class of data points by building mathematical models based on training data [4]. Computer-aided diagnosis (CAD) systems aim to assist clinicians and medical professionals in diagnosing diseases and conditions. For example, scholars are trying to design classifier-based computer models to diagnose Autism due to recent advances in ML. The CAD systems do not intend to make their diagnoses of patients. However, In the hands of clinicians, they can be valuable instruments for achieving an efficient diagnosis and faster diagnosis. Data preparation, dimension reduction, model training, validation, and testing are all aspects of ML pipelines. Pre-processing algorithms act as the front end of the pipeline. This can be attributed to the fact that they can improve some aspects of poor-quality data, namely outlier detection, feature scaling, and imputation, thus making the data more ready to be utilized further in the learning process [5]. Deep learning (DL) methods have recently gained popularity and have shown great potential in the medical field. More high-level features can be discovered with DL than with traditional ML methods. ML and image processing techniques have dramatically improved healthcare image processing and illness detection, achieving performance comparable to that of skilled specialists.

Deep learning is a powerful technique that simulates brain activity to create prototypes that can assist decision-making and data processing. Convolutional neural networks (CNNs), a deep learning model, are commonly used for analyzing visual images with minimal pre-processing. Machine learning (ML) as a diagnostic tool has become increasingly popular, providing additional information [6]. However, DL models are less reliable in clinical settings as they require large datasets and can be affected by the choice of hyperparameters. Transfer learning (TL) is a process that involves taking parts from one model and using them to build another model serving a different purpose. It can potentially improve DL models by enabling knowledge transfer between various tasks. Utilizing meta-learning for reuse may become more common in the future [7]. To improve performance, a process of optimization is used to select appropriate hyperparameter values rather than choosing them randomly [8].

Optimization is valuable in numerous fields, including engineering, mathematics, medicine, and the military. It involves selecting the best or most effective solution for a problem and improving its efficiency and effectiveness in the long run. An optimization process is an iterative approach that involves a thorough search of all possibilities to develop an ideal solution. There are two groups of literature optimization methods: deterministic and stochastic. Deterministic methods can achieve global optimum solutions within negligible error tolerance and converge in a finite amount of time but suffer from degraded performance in proportion to the size of the optimization problem. Stochastic optimization exploits the randomness of scenarios to probe searches superficially and can provide very efficient results despite not guaranteeing optimal results. Heuristic approaches such as evolutionary algorithms, nearest neighbor algorithms, memetic algorithms, insertion algorithms, and dynamic relaxations are less expensive and achieve near-optimal results. Still, most are tailored toward specific problems [[9], [10], [11], [12]].

There will likely be many layers, intermediate processing elements, and other structural elements in a proposed framework, meaning search metaheuristics will be needed to explore them. Metaheuristics, also known as stochastic algorithms, are a class of methods that provide efficient and reliable solutions to nonlinear optimization problems. A variety of metaheuristics solves optimization problems, and much of this intelligence comes from natural organisms in nature. Metaheuristics aims to offer a set of guidelines or rules for developing algorithms independent of the problem [10]. Despite the structural properties, metaheuristic approaches initiate arbitrary trials within their limits. Until the termination condition is met, each algorithm-specific equation evolves potential solutions.

Metaheuristic optimization involves two main phases - diversification and intensification - to find an optimal solution. Diversification is the exploration phase, which uses randomized searches to reduce the chance of being entrapped by local minima and maintain a global search. Intensification is the exploitation phase, which concentrates successful samples near the population memory to identify promising regions near the best solution. Balancing these two phases is crucial for successful metaheuristic optimization. Nature-inspired algorithms are commonly used for optimization problems, with examples of physics-based, nature-based, humans-based, swarm-based, and animal-based methods. Most metaheuristic algorithms are inspired by animal hunting and prey behavior, with three common types being evolutionary, physics, and swarm algorithms. Swarm algorithms simulate population behavior, with examples such as particle swarm optimization (PSO), ant colony optimization (ACO), and artificial bee colony algorithms. Other examples of swarm intelligence include firefly, gray wolf, Gorilla Troops Optimizers, and whale optimization algorithms [12].

Despite the existence of several algorithms for ASD detection, these algorithms fail to provide exact solutions to NP-hard multidimensional problems. This paper aims to fill that gap by introducing a novel deep-learning framework using TL with a Gorilla Troops optimizer for detecting ASD. The main contributions of this study can be organized as follows.

- Based on pre-trained CNNs, an innovative Autism Spectrum Disorders Detection via Transfer Learning with Gorilla Troops Optimizer (ASD^2^-TL∗GTO) framework has been devised.
- Gorilla outperformed natural-inspired algorithms among the most recent top algorithms.
- The ASD^2^-TL∗GTO framework is flexible; hyperparameters are not manually assigned.
- Two separate datasets are used, which makes the ASD^2^-TL∗GTO framework's deployment and data availability easier.
- Standard performance measurements have yielded extremely good outcomes.

The rest of the paper is organized as follows. First, the related work is reviewed in Section 2. Then, Section 3 presents the proposed ASD^2^-TL∗GTO framework. Next, Section 4 describes the experimental result. Section 5 wraps up this paper and discusses further research.

## 2 Related studies

Many fields of medicine, including structural and functional neuroimaging, are experiencing an expansion in the use of ML algorithms and DL approaches. Several neuroimaging studies have been conducted over the past few years to capture and analyze brain activity, including electroencephalography (EEG), magnetic resonance imaging (MRI), functional magnetic resonance imaging (fMRI), resting-state functional magnetic resonance imaging (rsfMRI), positron emission tomography (PET), and electrocorticography (ECoG) [[13], [14], [15], [16], [17]]. The following section discusses different techniques and neuroimaging studies proposed for ASD identification.

### 2.1 Machine learning for ASD identification

Many researchers have applied ML techniques for ASD classification [[17], [18], [19], [20]]. An automated postural control pattern detection algorithm using ML was developed and validated on the COP dataset for identifying children with Autism. Several supervised ML techniques were used to determine the ASD postural control. According to the findings, all ML algorithms successfully recognized postural control patterns between typically developing children and children with ASD [4]. On the other hand, physical activity levels in the ASD and typical development groups were not closely observed. Bilgen et al. [5] considered T1-weighted MRI data for the brain for autism spectrum disorders. This study investigated the link between brain area morphology and spatial representation using an ML-based diagnostic technique.

Nevertheless, the model has not proved accurate enough for ASD identification. Therefore, several ML methods and DL approaches such as Naïve Bayes (NB), support vector machine (SVM), Logistic regression (LR), K-nearest neighbor (KNN), and convolutional neural network (CNN) are implemented on the UCI dataset to analyze features and predict autism symptoms in children [21]. The results illustrate that CNN and SVM achieve the highest accuracy. An ensemble learning method is presented to represent deep features of the brain obtained from functional MRI (fMRI). This study used a stacked denoising autoencoder (SDA) to derive deep feature representation from multi-atlas images. To solve the task of ASD classification, multi-layer perceptron (MLP) and ensemble learning methods are employed. The proposed model exhibited an accuracy of 74.52 % [22]. Another study combined convolutional networks based on multi-atlas graphs and ensemble learning to diagnose ASD automatically [23]. A dataset of 949 subjects is used to evaluate the proposed approach, including 419 patients with ASD and 530 patients with typical control (TC). Chaitra et al. [24] combined graph-theoretic techniques with a support vector machine for ASD identification for 432 ASD patients. However, the experimental results reveal that the model could only diagnose ASD with 70.1 % accuracy.

### 2.2 Deep learning for ASD identification

Deep learning-based classification techniques have recently attracted much attention from researchers due to their ability to identify features and diagnose ASD effectively [2,25] automatically. By analyzing the brain activity patterns of patients, Heinsfeld et al. captured autism symptoms from a large brain imaging dataset [26]. The maximum accuracy of the model was 70 % based on rs-fMRI data. In a study by Ari et al. [27], EEG signals were used to diagnose high-risk Autism in children. The model's architecture consists of a sparse coding-based feature mapping (SCFM) algorithm, Douglas-Peucker (DP) approach, and CNNs. Initially, the DP algorithm decreases the EEG signal by reducing the number of samples for each channel. The Wavelet-derived EEG signals are then encoded using SCFM. In addition, extreme learning machines (ELM)--based autoencoders (AE) are utilized to improve CNN models' performance. The results of the experiment showed that the model was 98.88 % accurate. However, this study includes small samples; only 20 children with Autism were used for ASD classification.

Xu et al. examined the inferior frontal gyrus and temporal lobe abnormalities among 47 children with ASD using short-term memory networks (LSTMs) with attention mechanisms. ASD was classified with a high level of accuracy, with a specificity of 97.5 % [28]. However, sophisticated algorithms are needed to identify and capture high-level information from the fNIRS data. Moreover, a novel cognitive learning method based on long short-term memory and autoencoder networks was developed to investigate untraditional brain characteristics and capture ASD symptoms [29]. Enhanced convolutional neural networks (ECNNs) are also proposed to identify specific patterns to diagnose ASDs by analyzing functional connectivity patterns between different brain areas [30]. According to experimental results, the proposed ECNN can achieve 80 % classification accuracy. In Epalle et al.'s study, multi-input DL networks were used to classify autism symptoms. The architecture of the proposed model incorporates three different atlases to pre-process the neuroimaging data. In addition, the Hinge loss function was utilized for training the proposed DL network. As a result, the model reached a classification accuracy of 78.07 % [31].

Elbattah et al. [32] presented a novel application of transfer learning for ASD detection using eye-tracking. They employed transfer learning models such as VGG-16, ResNet, and DenseNet [33] for Autism diagnosis. These models comprise a base model for feature extraction and a classifier model for classification. Eye-tracking scan paths are converted into a visual representation to facilitate the use of pre-trained vision models. However, the study acknowledges that their review of potential machine-learning approaches for autism detection is confined to facial expressions and eye-gaze movements, potentially overlooking other significant features or modalities. Moreover, the small sample size used in the experiments may limit the generalizability of the results.

In summary, the literature indicates that many researchers use neuroimaging modalities, such as fMRI and rsfMRI, to detect Autism. Table 1 summarizes the related work for ASD. However, three main limitations can be concluded. First, detecting ASDs using EEG has traditionally been accomplished using traditional machine learning algorithms. EGG has shown superiority over other neuroimaging methods in terms of high temporal resolution, convenience, noninvasive nature, general availability for physicians, and low setup costs. Second, a few DL-based studies have been suggested to capture Autism using EGG [27]. Third, NP-hard multidimensional problems cannot be solved by these existing algorithms. This paper proposes a novel ASD^2^-TL∗GTO framework based on TL and an Artificial GTO for detecting Autism to fill the gap.

**Table 1:** Summary of previous studies in the field.

Reference	Dataset	Subjects	Model	Neuroimaging Modalities	Accuracy
Wang et al. [23]	ABIDE I	HC = 530 ASD = 419	- Multi-atlas graphs convolutional network	fMRI	75.86 %
Kashef et al. [30]	ABIDE I	HC = 21 ASD = 22	- CNN	rs-fMRI	80.0 %
Ari et al. [27]	Clinical Dataset from King Abdulaziz University Hospital	ASD = 20	- Douglas-Peucker (DP) algorithm - CNN	EEG	98.99 %
Xu et al. [28]	ABIDE I	HC = 21 ASD = 22	- Deep learning model - Attention mechanism	rs-fMRI	97.5 %
Liu et al. [29]	ABIDE I	HC = 352 ASD = 322	- LSTM - Autoencoder network - Attention mechanism	rs-fMRI	74.7 %
Epalle et al. [31]	ABIDE I	HC = 556 ASD = 432	- Multi-input deep neural network model	fMRI	78.07 %
Li et al. [4]	COP Dataset	ASD = 50	- Automated postural control pattern detection algorithm - Six Machine Learning Methods	EEG	90 %
Bilgen et al. [5]	ABIDE I	HC = 530 ASD = 419	- Machine learning-based models	T1-weighted MRI	70 %
Raj and Masood et al. [21]	UCI dataset	ASD = 704	- SVM, Nave Bayes, LR, and CNN	Not Mentioned	99.53 %
Wang et al. [22]	ABIDE I	HC = 530 ASD = 419	- Ensemble learning - Stacked Denoising Autoencoder - Multi-atlas graphs	fMRI	74.52 %
Chaitra et al. [24]	ABIDE I	HC = 556 ASD = 432	- Graph-theoretic techniques - SVM	fMRI	70.1 %
Heinsfeld et al. [26]	ABIDE I	HC = 556 ASD = 432	- Different deep learning methods	rs-fMRI	70.0 %

## 3 Methodology

This study proposes an ASD^2^-TL∗GTO framework for Deep Learning (DL) and Machine Learning (ML) classification and optimization, leveraging the Artificial Gorilla Troops Optimizer (GTO) metaheuristic optimizer. The GTO was chosen for its proficiency in efficiently exploring complex, high-dimensional search spaces and balancing exploration and exploitation during optimization. Inspired by the social behavior and intelligence of gorilla troops in the wild, the GTO is a novel metaheuristic optimization algorithm. It has demonstrated superior performance over other metaheuristic optimization algorithms across various optimization tasks and has been successfully applied to various optimization problems in diverse fields.

In the context of this study, we chose to use the Gorilla Troops optimizer in conjunction with transfer learning because of its ability to effectively optimize the weights of deep neural networks, which are widely used in transfer learning. Transfer learning involves reusing pre-trained models such as AlexNet [34], DenseNet [33], and MobileNet [35] to improve the performance of new models on different tasks, and the optimization of these models is a crucial step in achieving high performance. The Gorilla Troops optimizer effectively optimizes the weights of deep neural networks, making it well-suited for use in transfer learning applications.

**Fig. 1:**
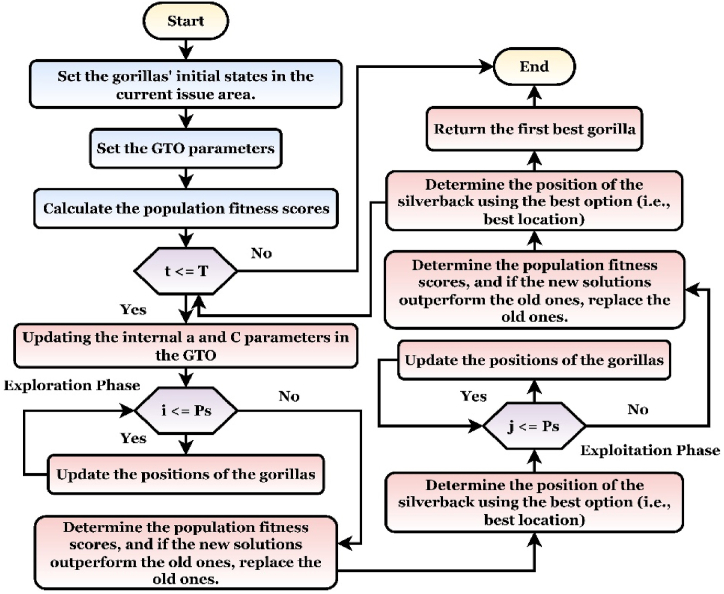
Flowchart illustrating the process of the gorilla troops optimizer (GTO) algorithm.

The flowchart of the GTO algorithm is depicted in Fig. 1. This section introduces the Artificial Gorilla Troops Optimizer (GTO) metaheuristic optimizer, followed by a detailed discussion of the proposed framework's internal components. The framework comprises four phases, beginning with data collection. The current study utilizes two distinct datasets, one numerical and the other comprising images. These datasets are then pre-processed to suit the subsequent classification and optimization stage better. Following this, the initial GTO population is generated. The pre-processed datasets and the GTO population are then employed in the classification and optimization phase.

### 3.1 Artificial Gorilla Troops Optimizer (GTO)

GTO is a metaheuristic optimization algorithm rooted in the gorillas' lifestyle. GTO (gorilla behavior optimization) was proposed by Abdollahzadeh et al., in 2021. Simulation of gorilla social behavior and movement [36,37] is achieved through the technique. The gorilla troops comprise a silverback troop and a family of females and their offspring. Other groups of male gorillas also exist. A silverback lives for about 12 years and gets its name from the silvery hair that grows on its back during puberty. Members of this group revolve around Silverback.

Additionally, it dictates the group's movement, determines whether they fight, ensures their safety, and directs them to food sources. Young male gorillas, known as blackbacks, provide backup protection for silverbacks. Their backs are not covered in silver hair; they are between 8 and 12 years old. The male gorillas migrate from their birthplaces as well as the females. New groups of gorillas usually form from these migrations. It is common for male gorillas to break away from their group and form one with a female who has recently moved out. Male gorillas may remain in the community where they were brought up and are referred to as silverbacks. Certain gorillas may compete with or continue to lead the team to achieve their goals without the Silverback when the Silverback dies [36,37].

The GTO optimizer showed high accuracy and efficiency [38]. This optimizer requires little adjustment for engineering applications [38], as it is easy to use. Additionally, by enhancing search capabilities, the GTO method may be used to explore other system dimensions. When the dimensions are increased, the performance of other optimizers declines noticeably, giving this one an advantage in all similar dimensions [38]. Gorillas prefer to live in groups, so they cannot live alone.

Consequently, the gorillas hunt together for food and remain under a silverback leader who makes all the group's decisions. A silverback is considered the best gorilla in this algorithm, and all others tend to approach it, while the weakest is ignored since it is the least preferred. The gorilla is represented by X in this algorithm, while GX represents silverbacks. Take a gorilla that is seeking better food sources, for example. Thus, GX is generated each time an iteration occurs and exchanges with the next solution if an improved value can be found [36]. Moreover, the algorithm can be divided into two phases, as illustrated below.

### 3.2 GTO exploration phase

GTO's gorilla optimization algorithms consider silverbacks the best candidate solutions for each optimization operation stage, and all gorillas are considered contenders. Three operators have conducted exploration: migration to unknown areas to increase GTO exploration. By moving to other gorillas, the second operator achieves a balance between exploring and exploiting. GTO can significantly improve their search for different optimization spaces by using the third operator during the exploration phase, transfer towards a known and effective space. A parameter named p can be used to select the migration mechanism for an unknown position. The attribute p in the scope of [0,1] must be provided before executing the optimization process to determine the likelihood of selecting a transition plan to an unknown position [39]. If rand<p, then the first mechanism is selected. In the case of rand≥0.5, however, the mechanism is selected to move toward other gorillas. If rand<0.5, The decision is made to migrate to a known location. Any mechanism can help the algorithm perform well, depending on the mechanism utilized. All results are evaluated at the end of the exploration phase, and ${GX}^{\left(t\right)}$ is used instead of $X^{\left(t\right)}$ (Silverback) if it is the lowest-cost option.

### 3.3 GTO exploitation phase

This phase can be divided into two processes: Following the Silverback and Competition for adult females. First, the value of D can be used to decide. D is calculated in the Equations below with the randomly selected variable W, starting the optimization process [38].

#### 3.3.1 Follow the silverback

Silverback, the leader of the newly formed group, is a young, healthy male whom the other gorillas closely watch. In the same way, they follow Silverback's instructions to collect food and explore different areas. In addition, members of the group can affect movement within the group. Using this strategy when D≥W, Silverback commands his gorillas to search for food from different food sources.

#### 3.3.2 Competition for adult females

At puberty, teenage gorillas compete for female members of their group with other males, a process that is usually violent. However, this strategy is applicable in situations when D<W. As a result, if the cost of ${GX}^{\left(t\right)}$ is lower than the cost of $X^{\left(t\right)}$, then ${GX}^{\left(t\right)}$ replaces $X^{\left(t\right)}$, and is a better alternative (Silverback) [39].

### 3.4 Data acquisition and description

The datasets in this study are acquired from two public sources: Kaggle and UCI ML Repository. The first dataset is the Autistic Children Data Set, consisting of 3,374 images partitioned into “Autistic” and “Non-Autistic” cases [40]. The Autistic Children Dataset consists of facial images of children. The second dataset is merged from three numerical repositories: (1) Autism Screening Adult, which consists of 704 records [41]; (2) Autistic Spectrum Disorder Screening Data for Adolescents, which consists of 104 records [42]; and (3) Autistic Spectrum Disorder Screening Data for Children that consists of 292 records [43]. They consist of 21 attributes. After the merge process, the second dataset consists of 1,100 records; five samples are shown in Table 2. It shows that the first ten columns consist of Boolean values. There are another three numeric attributes and eight categorical attributes.

**Table 2:** Samples from the second merged dataset.

A1	A2	A3	A4	A5	A6	A7	A8	A9	A10	Age	G	Ethnicity	Jaundice	Family Member with PDD	Residence	Used Screening App	Result	Age Desc. (years)	Who is Completing the Test?	Class
1	1	0	0	1	1	0	1	0	0	6	m	Others	no	no	Jordan	no	5	4–11	Parent	NO
1	1	0	0	1	1	0	1	0	0	6	m	Middle Eastern	no	no	Jordan	no	5	4–11	Parent	NO
1	1	0	0	0	1	1	1	0	0	6	m	?	no	no	Jordan	yes	5	4–11	?	NO
0	1	0	0	1	1	0	0	0	1	5	f	?	yes	no	Jordan	no	4	4–11	?	NO
1	1	1	1	1	1	1	1	1	1	5	m	Others	yes	no	United States	no	10	4–11	Parent	YES

The datasets utilized in this study are publicly accessible and have been anonymized. The authors, however, have limited knowledge regarding the specific process of classifying facial images. It is presumed that the contributors of the dataset annotated the images, with medical professionals such as clinicians and doctors likely involved in the diagnosis and classification process.

The Autistic Children Data Set used in our study comprises facial images of children, classified based on a known diagnosis. The specifics of the classification process are not accessible to us, as the dataset was procured from a public source, Kaggle. It is believed that the images were annotated by the individuals who uploaded the dataset, with the involvement of clinicians and doctors in the diagnosis and classification of the cases.

The Autistic Children Data Set contains 3,374 facial images of children. The second dataset compiles data from three numerical repositories, encompassing 21 screening attributes. It's important to note that these datasets were obtained from public sources - Kaggle and the UCI ML Repository. All data used in our study are anonymized and publicly available.

### 3.5 Data pre-processing

The data pre-processing phase readies the datasets for the subsequent classification and optimization stages. As the data acquisition phase outlines, this study employs two different datasets (i.e., images and numerical records), necessitating distinct pre-processing techniques. The images in the Autistic Children Data Set vary in size; therefore, they are resized to a uniform dimension of (128, 128, 3). Given that this dataset is balanced, there is no need for data-balancing techniques at this stage. The numerical dataset contains categorical attributes, which are transformed using label encoding. This method converts categorical labels into numerical ones. Cells with question marks representing unanswered entries are replaced with zeros.

Five data scaling techniques are utilized in the current study for the images. They are (1) normalization, (2) standardization, (3) min-max scaling, (4) max-abs scaling, and (5) robust scaling. The equations behind them are shown from Equation (1) to Equation (5), respectively, where $X_{i}$ is the input record, $X_{0}$ is the scaled output record, μ is the record mean, σ is the record standard deviation, $Q_{1}$ is the first quartile, and $Q_{3}$ is the third quartile.

(1) $$X_{0}=\frac{X_{i}}{\max\left(X\right)}$$

(2) $$X_{0}=\frac{X_{i}-\mu }{\sigma }$$

(3) $$X_{0}=\frac{X_{i}-\min\;\left(X_{i}\right)}{\max\;\left(X_{i}\right)-\min\left(X_{i}\right)}$$

(4) $$X_{0}=\frac{X_{i}}{\left|\max\;\left(X_{i}\right)\right|}$$

(5) $$X_{0}=\frac{X_{i}-Q_{1}\left(X_{i}\right)}{Q_{3}\left(X_{i}\right)-Q_{1}\left(X_{i}\right)}$$

### 3.6 GTO initial population generation

This study utilizes the GTO optimization for both datasets. The GTO is used with the first dataset and DL CNN models to find the best models' hyperparameters, while it is used with the second dataset and machine learning models to select the most promising features. However, the initial population generation for them is the same. The population is randomly generated, and the population size is set to $N_{\max}$. Each solution is a vector of size (1×D) in the population, where each element is in the range ∈[0,1]. The value of D is determined concerning the dataset. In other words, D will equal the number of hyperparameters for the first dataset and the number of attributes for the second dataset. Equation (6) illustrates the initialization process of the population matrix, where population represents the whole population matrix, LB, and UB represent lower and upper boundaries, and rand represents random values ∈[0,1].

(6) Population=rand×(UB−LB)+LB

### 3.7 Classification and GTO optimization phase

**Fig. 2:**
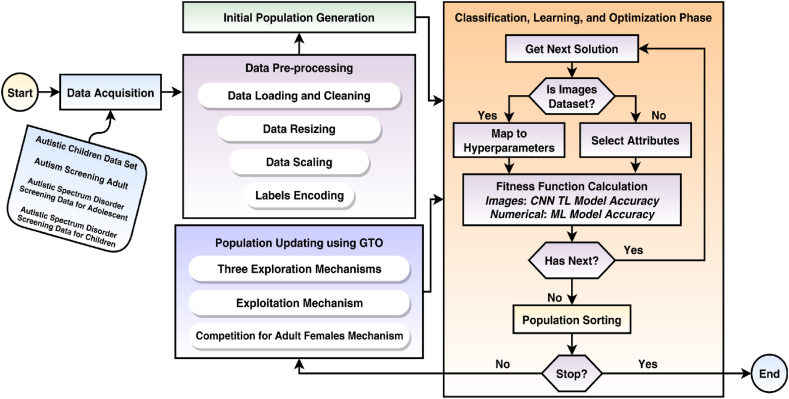
Proposed autism detection framework integrating deep learning, machine learning, and gorilla troops optimizer (GTO).

The learning phase begins when the datasets have been pre-processed and the initial population has been created. In this phase, various transfer learning hyperparameters, such as data augmentation and batch size, are optimized using the GTO metaheuristic optimizer. For each pre-trained transfer learning model being utilized, the goal is to determine the best hyperparameter values. There are three processes involved in this step. These are summarized in Fig. 2. The first and second phases in the Figure run once, while T[max] repeatedly iterates for the other two phases.

#### 3.7.1 Fitness score calculation

In this step, the fitness function score for each solution is evaluated in relation to the given dataset. As previously mentioned, each solution cell contains a random value ∈ [0,1]. Therefore, mapping these floating numbers to corresponding values is necessary for each dataset. For the image dataset, the cell values are mapped to hyperparameters, as outlined in Table 3. For the numerical dataset, the cell values are mapped to Boolean flags, determining whether to retain or drop the columns of the features.

**Table 3:** Hyperparameters for optimization using transfer learning and image dataset.

Cell Index	Hyperparameter Definition
1	Loss function
2	Batch size
3	Dropout ratio
4	TL learning ratio
5	Parameters (i.e., weights) optimizer
6	Scaling technique
7	Apply data augmentation (DA) or not.
8	Rotation value (if DA is applied)
9	Width shift value (if DA is applied)
10	Height shift value (if DA is applied)
11	Shear value (if DA is applied)
12	Zoom value (if DA is applied)
13	Horizontal flipping Flag (if DA is applied)
14	Vertical flipping flag (if DA is applied)
15	Brightness changing range (if DA is applied)

*How to map from the cells' values to hyperparameters?* To understand how this mapping procedure works, consider mapping the batch size from the solution cell to an associated hyperparameter. The range of batch sizes from which to choose must first be determined. This paper utilizes the “4 → 48 (step = 4)" range. This results in twelve possible outcomes. Equation (7) can be used to calculate the possibility. For Instance, suppose the random numeric value is 0.75, and the possibilities are 12, then the index is 9. (i.e., the batch size value of 36). In Table 4, the ranges of hyperparameters are shown. The target pre-trained transfer learning model is assembled after mapping each element in the solution to the associated hyperparameter. This study's pre-trained transfer learning CNN models include DenseNet169, DenseNet201, MobileNet, MobileNetV2, MobileNetV3Small, and MobileNetV3Large with the “ImageNet” pre-trained weights [44,45]. In the current investigation, each pre-trained transfer learning CNN model will start learning on the split subsets for several epochs equal to 5. The generalization of the pre-trained transfer learning CNN model is tested on the entire dataset.

(7) RangeIndex=⌈solution[index]×Length(ranges[index])⌉

*How to map from the cells' values to a new data subset?* Equation (8) maps from the cells' values to Boolean flags to keep or drop the features' columns. For the cell_*i*_ at index i, if its value is greater than or equal to 0.5, the features' column is kept and dropped otherwise. The ML models used in this study include Decision Tree (DT), Extra Trees (ET), and Light Gradient Boosting Model (LGBM). The grid search is applied to fetch the best ML models' hyperparameters. There are two hyperparameters for the DT and ET (i.e., criterion and splitter). The splitter has the options best and random, while the criterion has the options Gini and entropy. The LGBM has a single hyperparameter (i.e., learning rate) with the values [0.01*,*0.1*,*1.0]. The number of estimators is fixed at 300. Cross-validation is used in the ML models for five folds.

(8) $${State}_{i}=\left\{ \begin{array}{c} {Keep\;Feature}_{i}if\left(Cell\geq 0.5\right)\\ {Drop\;Feature}_{i}Otherwise \end{array}\right.$$

how is performance evaluated? Various performance metrics, such as accuracy, Area Under the Curve (AUC), and specificity, are computed to assess the model's performance. Specifically, accuracy is calculated by dividing the number of correct predictions by the total sample count. Sensitivity, or recall, indicates the proportion of actual positive samples correctly identified, reflecting the classifier's ability to detect positive instances correctly. On the other hand, specificity deals with truly negative samples, indicating the proportion of actual negative samples correctly identified. Precision represents the proportion of true positives among all instances classified as positive. The F1-score is a harmonic mean of precision and recall, providing a balanced measure of these two metrics. The AUC intuitively evaluates the overall quality of the classifier. In this study, we employ the following performance metrics: Accuracy (Equation (9)), Precision (Equation (10)), Specificity (Equation (11)), Recall (or Sensitivity) (Equation (12)), Area Under Curve (AUC), Intersection over Union (IoU), Dice (Equation (13)), Cosine Similarity, F1-score (Equation (14)), Youden Index (Equation (15)), Balanced Accuracy, and Overlap Index.

(9) $$Accuracy=\frac{TP+TN}{TP+TN+FP+FN}$$

(10) $$Precision=\frac{TP}{TP+FP}$$

(11) $$Specificiy=\frac{TN}{TN+FP}$$

(12) $$Recall=Sensitivity=\frac{TP}{TP+FN}$$

(13) $$Dice\;Coef=\frac{2\times T_{P}}{{2\times T}_{P}+F_{P}+F_{N}}$$

(14) $$F1-Score=\frac{2\times Precision\times Recall}{Precision+Recall}$$

(15) YoudenIndex=Specificity+Sensitivity−100%

**Table 4:** Configurations of the conducted experiments.

Configuration	Which Dataset?	Specifications
Apply Dataset Shuffling?	Both	Yes (Random)
Metaheuristic Optimizer		Artificial Gorilla Troops Optimizer (GTO)
Scripting Language		Python
Python Main Packages [46]		(1) Tensor ow, (2) Keras, (3) Sklearn, (4) NumPy, (5) OpenCV, and (6) Matplotlib
Working Environment		Google Colab + GPU
a Solutions (Population)		10
a Iterations		25
Scalers		(1) Normalization, (2) Standardization, (3) Min-Max Scaling, (4) Max-Absolute Scaling, and (5) Robust Scaling
Image Size	First	(128 × 128 × 3)
Train Split Ratio		80 %–20 % (i.e., 80 % for training and validation and 20 % for testing)
a Epochs		5
Output AF		SoftMax
Pre-trained Models		(1) DenseNet169, (2) DenseNet201, (3) MobileNet, (4) MobileNetV2, (5) MobileNetV3Small, and (6) MobileNetV3Large
Pre-trained Weights Initializers		ImageNet
Losses		(1) Categorical Crossentropy, (2) Poisson, (3) KLDivergence, (4) Categorical Hinge, (5) Squared Hinge, and (6) Hinge
Weights Optimizers		(1) Adam [47], (2) NAdam, (3) AdaMax, (4) AdaDelta, (5) AdaGrad, (6) RMSProp, (7) Ftrl, (8) SGD, (9) SGD Nesterov, (10) RMSProp Centered, and (11) Adam AMSGrad
Dropout Range		[0 → 0.6]
Batch Size Range		4 → 48 (step = 4)
Pre-trained MLR Range		1 → 100 (step = 1)
Apply DA?		Yes or No
DA Rotation Range		0° →45 ° (step = 1 °)
DA Width Shift Range		[0 → 0.25]
DA Height Shift Range		[0 → 0.25]
DA Shear Range		[0 → 0.25]
DA Zoom Range		[0 → 0.25]
DA Horizontal Flip Range		Yes or No
DA Vertical Flip Range		Yes or No
DA Brightness Range		[0.5 → 2.0]
Grid Search	Second	True
K-fold CV		5
ML Models		(1) Decision Tree (DT), (2) Extra Trees (ET), and (3) Light Gradient Boosting Model (LGBM)

a Number of, DA: Data Augmentation, ML: Machine Learning, MLR: Model Learn Ratio, CV: Cross Validation, AF: Activation Function.

#### 3.7.2 Population updating

The population is ordered in descending order according to fitness ratings. Consequently, the top choice is the best, and the bottom choice is the worst. This step is necessary to determine whether $X_{\text{best}}^{t}$ and $X_{\text{Worst}}^{t}$ are required for the population update process. The GTO metaheuristic optimizer determines the ideal hyperparameters for each CNN model. Three steps make up the GTO's operation. They are (1) three mechanisms for exploration, (2) one mechanism for exploitation, and (3) one mechanism for competing for adult females. Equation (16) represents an expanded exploration process; the exploitation mechanism is presented in Equation (17). Equation (18) presents the mechanism for competition for adult females, where $r_{1}$, $r_{2}$, and $r_{4}$ are three random values, $X_{r}^{\left(t\right)}$ represents a random solution from the population; a silverback gorilla position vector (i.e., best solution) is $X_{silverback}$, the impact force is mimicked by Q, and the coefficient vector A represents the level of violence in a conflict [48].

(16) $$X_{{GTO}_{1}}^{\left(t+1\right)}=\left\{ \begin{array}{c} LB+r_{1}\times \left(UB-LB\right),if\;\left(rand<p\right)\\ L\times H+\left(r_{2}-C\right)\times X_{r}\left(t\right),if\;\left(rand\geq 0.5\right)\\ X\left(i\right)-L\times (L\times \left(X^{\left(t\right)}-X_{r}\left(t\right)\right)+r_{3}\times \left(X^{\left(t\right)}-X_{r}^{t}\right),Otherwise \end{array}\right.$$

(17) $$X_{{GTO}_{2}}^{\left(t+1\right)}=L\times M\times \left(X^{\left(t\right)}-X_{silverback}\right)+X^{\left(t\right)}$$

(18) $$X_{{GTO}_{2}}^{\left(t+1\right)}=X_{silverback}-\left(X_{silverback}\times Q-X^{\left(t\right)}\times Q\right)\times A$$

### 3.8 The suggested framework overall pseudocode

The steps are computed iteratively for a maximum number of iterations $T_{\max}$. After that, the best combination can be used in any subsequent analysis. The suggested overall classification, learning, and hyperparameters optimization strategy is summarized by Algorithm 1.

**Table:** Algorithm 1: Pseudocode for the Proposed ASD2-TL∗GTO Framework

	**Input:***classifier*, *dataset*			//Target Model, Used Dataset
	**Output:***bestScore, bestSolution*			//Best score and best solution
*1*	*X* = RandomPopulationGeneration()			//Create the initial solutions.
	//Use the GTO metaheuristic optimizer to carry out the learning hyperparameters optimization procedure for $T_{\max}$ iterations			
*2*	*t* = 1s			//Initialize the iterations' counter where $t\leq T_{\max}$.
*3*	**while** ($t\leq T_{\max}$) do			//Make a score calculation for each solution.
*4*	i=1			//Initialize the counter where $i\leq N_{\max}$
*5*	scores=[]			//Initialize the scores list.
*6*	**while** ($i\leq N_{\max}$) do			
*7*		**if** (Is Images Dataset?), **then**		
*8*			classifier = **CreatePretrainedCNNModel**()	//Create the pre-trained TL model.
*9*			boolX = **MapSolutionValuesToBooleans**(X[i])	//Transform random numbers into hyperparameters.
*10*			score = **FitnessScoreCalculation (**classifier, boolX, dataset)	//Determine the current solution's fitness score.
*11*		Else		
*12*			classifier = **CreateMachineLearningModel** ()	//Create the ML model.
*13*			boolX = **MapSolutionValuesToBooleans** (X[i])	//Convert the values into keep-or-drop Boolean flags.
*14*			subset = **ExtractDataSubset**(boolX, dataset)	//Extract the new subset.
*15*			score = **FitnessScoreCalculation** (classifier, boolX, dataset)	//Determine the current solution's fitness score.
*16*		**Append**(score, scores)		//Add the result to the list of scores.
*17*		i=i+1		//Increase counter by 1.
	//GTO metaheuristic optimizer is used to update the population.			
*18*		X = **UpdateSolutionsViaGTO**(X, scores)		
		//Get the current iteration's best score and best solution.		
*19*	bestScore, bestSolution = **GetBest**(X, scores)			
*20*	t = t +1			//Add 1 to the iterations counter.
*21*	return **bestScore, bestSolution**			//Return the best score and the best solution

## 4 Experiments and discussions

The experiments' results and analyses are presented in the current section. The common configurations used in the experiments for both datasets are listed in Table 4.

### 4.1 The “Autistic Children Data Set” experiments

**Fig. 3:**
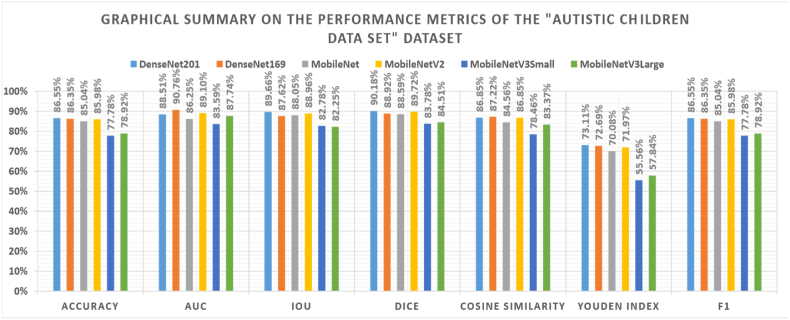
Visual summary of performance metrics for the autistic children dataset.

For the first dataset, the best hyperparameters are presented in Table 5, while the corresponding performance metrics are presented in Table 6. Fig. 3 summarizes the best performance metrics for each used TL model graphically. The measurements are displayed on the x-axis, while the scores are on the y-axis. They show that a TF learning ratio above or equal to 50 % is recommended. Using categorical cross-entropy loss function is recommended by three models.

**Table 5:** Optimal hyperparameters for pre-trained CNN models applied to the autistic children dataset.

Model Name	DenseNet201	DenseNet169	MobileNet	MobileNetV2	MobileNetV3Small	MobileNetV3Large
TF Learn Ratio	50 %	77 %	89 %	8 %	52 %	87 %
Loss	Squared Hinge	KLDivergence	Categorical Crossentropy	Categorical Crossentropy	Squared Hinge	Categorical Crossentropy
Batch Size	16	40	44	24	4	8
Dropout	0.27	0.22	0.6	0.13	0.6	0
Optimizer	SGD	SGD Nesterov	RMSprop Cantered	SGD	SGD	SGD
Scaling Technique	Robust	Standardization	Normalization	Max-Abs	Robust	Robust
Apply Augmentation	Yes	Yes	Yes	Yes	Yes	Yes
Rotation Range	3	41	44	7	27	44
Width Shift Range	0.08	0.19	0.06	0.07	0.01	0.19
Height Shift Range	0.23	0.11	0.25	0.11	0.21	0.04
Shear Range	0.22	0.02	0.15	0.12	0.09	0.21
Zoom Range	0.22	0.16	0.17	0.17	0.23	0.1
Horizontal Flip	Yes	Yes	No	Yes	Yes	Yes
Vertical Flip	Yes	No	Yes	No	Yes	No
Brightness Range	1.38–2.0	1.23–1.37	1.91–2.0	0.82–1.23	1.59–1.7	0.65–1.7

**Table 6:** The best performance metrics reported by the Autistic Children Dataset.

Model Name	DenseNet201	DenseNet169	MobileNet	MobileNetV2	MobileNetV3Small	MobileNetV3Large
Loss	0.777	0.512	0.965	0.874	0.950	0.533
Accuracy	86.55 %	86.35 %	85.04 %	85.98 %	77.78 %	78.92 %
AUC	88.51 %	90.76 %	86.25 %	89.10 %	83.59 %	87.74 %
IoU	89.66 %	87.62 %	88.05 %	88.96 %	82.78 %	82.25 %
Dice	90.18 %	88.92 %	88.59 %	89.72 %	83.78 %	84.51 %
Cosine Similarity	86.85 %	87.22 %	84.56 %	86.85 %	78.46 %	83.37 %
Youden Index	73.11 %	72.69 %	70.08 %	71.97 %	55.56 %	57.84 %
F1	86.55 %	86.35 %	85.04 %	85.98 %	77.78 %	78.92 %

Using the SGD parameters, the optimizer is recommended by four models. Three models recommend the robust scaling technique. Applying data augmentation is recommended by all models. Applying horizontal flipping is recommended by five models. The lowest loss value is 0.512, which the DenseNet169 model reports. The highest accuracy, AUC, IoU, dice, cosine similarity, and F1-score are 86.55 %, 90.76 %, 89.66 %, 90.18 %, 87.22 %, 73.11 %, and 86.55 %, respectively, by DenseNet201, DenseNet169, DenseNet201, DenseNet201, DenseNet169, DenseNet201, and DenseNet201 models respectively. From that, DenseNet201 and DenseNet169 are considered the best models.

### 4.2 The second merged dataset experiments

**Fig. 4:**
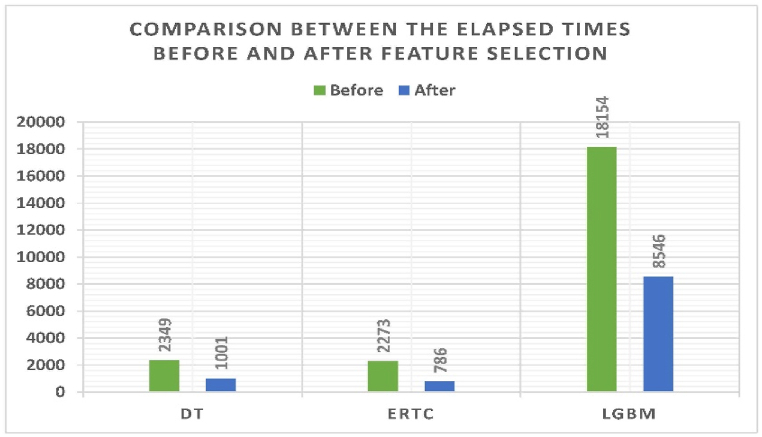
Graphical comparison of performance metrics before and after feature selection, with green bars representing ‘before’ and blue bars indicating ‘after'

The current study uses three numerical datasets, as discussed in Subsection 3.4. Table 7 shows the performance metrics before feature selection, while Table 8 shows the performance metrics after feature selection. They show that feature selection using GTO improved the performance metrics and decreased the elapsed time. They also show that five experiments recommend standardization, and the last five attributes are unnecessary in the classification process. Fig. 4 shows a graphical comparison between before and after using feature selection.

**Table 7:** Performance metrics before feature selection for the second dataset.

Classifier	DT	ERTC	LGBM
Elapsed time (ms)	2,349	2,273	18,154
Scaling Technique	Standardization	Robust Scaling	Standardization
TP	707	687	707
TN	393	370	393
FP	0	23	0
FN	0	20	0
Accuracy	100 %	96.09 %	100 %
Balanced Accuracy	100 %	95.66 %	100 %
Precision	100 %	96.76 %	100 %
Specificity	100 %	94.15 %	100 %
Recall	100 %	97.17 %	100 %
F1	100 %	96.97 %	100 %
Dice	100 %	96.97 %	100 %
Overlap Index	100 %	96.97 %	100 %
IoU	100 %	94.11 %	100 %
ROC	100 %	95.67 %	100 %
Youden Index	100 %	91.32 %	100 %

**Table 8:** Performance metrics following the feature selection process for the second dataset.

Classifier	DT	ERTC	LGBM
Elapsed time (ms)	1,001	786	8,546
Scaling Technique	Standardization	Standardization	Standardization
Encoded Solution	010100001000010100000	010100001001010100000	001100000010000000000
No of Ones	5	6	3
TP	707	707	707
TN	393	393	393
FP	0	0	0
FN	0	0	0
Accuracy	100 %	100 %	100 %
Balanced Accuracy	100 %	100 %	100 %
Precision	100 %	100 %	100 %
Specificity	100 %	100 %	100 %
Recall	100 %	100 %	100 %
F1	100 %	100 %	100 %
Dice	100 %	100 %	100 %
Overlap Index	100 %	100 %	100 %
IoU	100 %	100 %	100 %
ROC	100 %	100 %	100 %
Youden Index	100 %	100 %	100 %

Some potential limitations of the study could include the limited size and heterogeneity of the datasets used, which may affect the generalizability of the findings. Furthermore, the study focuses mainly on diagnostic accuracy without considering other relevant clinical outcomes, such as the impact of the proposed intelligent system on patient management and quality of life. Finally, this study does not address the issue of interpretability of the proposed framework, which is an essential aspect of medical diagnosis and rehabilitation.

## 5 Conclusions and future work

The study proposes a Deep Learning and Machine Learning-based intelligence system using the Artificial Gorilla Troops Optimizer (GTO) metaheuristic optimizer to detect autism spectrum disorders (ASD). The study uses two datasets, including images of Autistic and non-autistic children and numerical repositories. The study uses various data scaling techniques, and GTO is employed to determine optimum hyperparameters for deep learning CNN models with the first dataset. In contrast, it is utilized to find the best features with the second dataset and machine learning models. The proposed method has potential clinical applications to aid doctors in accurately diagnosing ASD, leading to more effective treatments and better patient outcomes. In terms of future research, the study plans to expand to include a larger dataset and investigate other transfer learning architectures. The proposed method also has the potential to be applied in other medical areas where the diagnosis is challenging, leading to improved medical decision-making and patient outcomes.

**Compliance with Ethical Standards:** Ethics Approval This article contains no studies with human participants or animals performed by authors. The authors certify that they have no affiliations with or involvement in any organization or entity with any financial or non-financial interest in the subject matter or materials discussed in this manuscript.

## Data availability statement

The data supporting this study's findings are.

• Senol, Cihan. “Autism children data set,” 2020. Accessed on October 19, 2023. Available at: https://www.kaggle.com/datasets/cihan063/autism-image-data.
• Fadi Fayez Thabtah. “Autism screening adult data set,” 2017. Accessed on October 19, 2023. Available at: UCI Machine Learning Repository - https://archive.ics.uci.edu/dataset/426/autism+screening+adult.
• “Autistic spectrum disorder screening data for adolescent data set,” 2017. Accessed on October 19, 2023. Available at: UCI Machine Learning Repository - https://archive.ics.uci.edu/dataset/420/autistic+spectrum+disorder+screening+data+for+adolescent.

## CRediT authorship contribution statement

**Abdulqader M. Almars:** Conceptualization, Data curation, Formal analysis, Funding acquisition, Investigation, Writing – original draft, Writing – review & editing. **Mahmoud Badawy:** Investigation, Methodology, Project administration, Resources, Software, Supervision, Validation, Visualization, Writing – original draft, Writing – review & editing. **Mostafa A. Elhosseini:** Methodology, Project administration, Resources, Software, Supervision, Validation, Visualization, Writing – original draft, Writing – review & editing, Formal analysis, Investigation.

## Declaration of competing interest

The authors declare that they have no known competing financial interests or personal relationships that could have appeared to influence the work reported in this paper.
